# Lovering’s Etherial Varnish

**Published:** 1862-10

**Authors:** 


### LOVERING'S ETHERIAL VARNISH.

This is a preparation for varnishing models for vulcanite work, the object of which is to prevent the plaster from adhering to, or rather being forced into, the rubber plate. This preparation accomplishes the objeet very perfectly. It saves much labor in finishing up the work. This preparation, or something of the kind, should alwaysbe used in the construction of rubber work.

It is for sale at Toland's dental depot. T.

